# Supplementary material for: Overexpression of DoBAM1 from Yam (Dioscorea opposita Thunb.) Enhances Cold Tolerance in Transgenic Tobacco
Source: Genes (Basel). 2022 Dec 6;13(12):2296. doi: 10.3390/genes13122296 (PMC9777697; doi:10.3390/genes13122296)
Supplement: Supplementary file 1 [file genes-13-02296-s001.zip › genes-1981653-supplementary.pdf]

Table S1. Related primer sequences of *DoBAM1* gene.

Primer type	Primer name	Prime sequence（5′→3′）
5′ RACE primers	DoBAM-5'R1	TCTATAAGCCCCCACCACACATCCAT
3′ RACE primers	DoBAM-3'F1	CTTAACCGATACGATAAGTACGCATA
ORF primers	DoBAM-ORF-F	ATGTTACACTGCGTTCATTC
	DoBAM-ORF-R	TACTTAAACAGGGAGATGAG
qRT-PCR primers	DoBAM-qF1	TATGACTGGACTGCTTACAGGAAGTT
	DoBAM-qR1	GCTGGTTATCCACTCCAAGGC
Transient expression vector primers	DoBAM-SF	CGGGGTACCATGTTACACTGCGTTCATTC
	DoBAM-SR	GCGGATCCTACTTAAACAGGGAGATGAG
Plant expression vector primers	DoBAM-ZF	GCGGATCCATGTTACACTGCGTTCATTC
	DoBAM-ZR	CGGGGTACCCTTAAACAGGGAGATGAGT
Inner control gene 18S rRNA primers	18S-F	CCATAAACGATGCCGACCAG
	18S-R	AGCCTTGCGACCATACTCCC
